# Plastic Debris in Agroecosystems: Distribution and Abundance Patterns, and Relationship with Terrain Characteristics in Southeastern Brazil

**DOI:** 10.1021/acsomega.4c10678

**Published:** 2025-05-01

**Authors:** John Jairo Arévalo-Hernández, Angela Barrera de Brito, Junior Cesar Avanzi, Marcelo Angelo Cirillo, Marx Leandro Naves Silva

**Affiliations:** † Federal University of Lavras, Department of Soil Science, P.O. Box 3037, Lavras 37203-202, Minas Gerais, Brazil; ‡ Surcolombiana University, Engineering Faculte, Avenida Pastrana Borrero, Carrera 1, Neiva 410001, Huila, Colombia; § Federal University of Lavras, Department of Physics, P.O. Box 3037, Lavras 37203-202, Minas Gerais, Brazil; ∥ Federal University of Lavras, Department of Statistics, P.O. Box 3037, Lavras 37203-202, Minas Gerais, Brazil

## Abstract

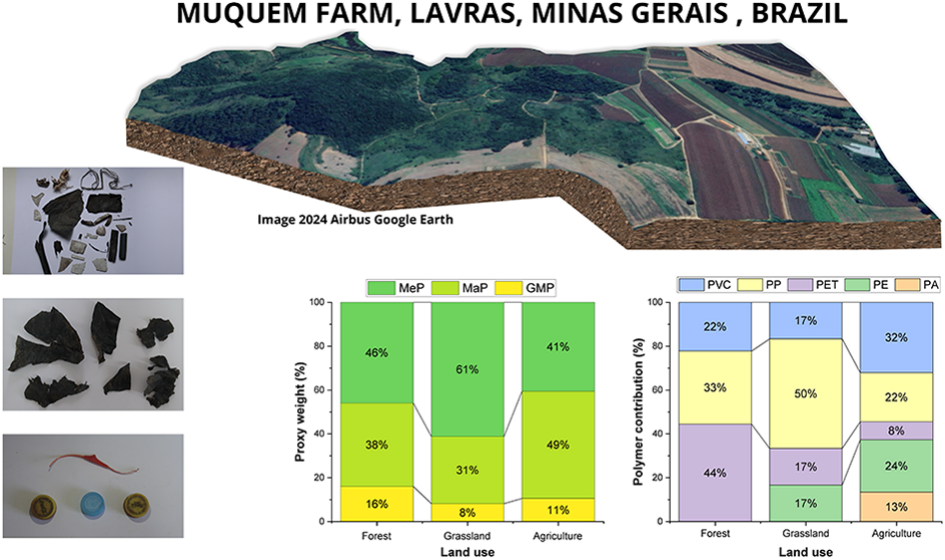

Plastic pollution in agricultural soil is a major concern, affecting soil biodiversity and functionality. In this context, studies of agricultural soil plastic pollution that consider its use across different regions are essential. Considering land use (forest, grassland, and agriculture), this study aimed to identify, quantify, and characterize plastic debris in various agroecosystems within a Southeast Brazil sub-basin. Additionally, the sampled plastic debris was georeferenced, allowing its characteristics to be correlated with terrain features, such as the LS factor and vegetation cover. Based on size, the plastic debris was categorized into macroplastics, mesoplastics, and coarse microplastics. The results revealed that agricultural areas accounted for 91.2% of the total plastic waste collected. The most common polymer types identified were polypropylene, polyethylene, and poly(vinyl chloride), comprising 82.6% of the total. The accumulation of plastic debris in this region was primarily linked to intensive human activity and agricultural practices. Moreover, its distribution strongly correlated with terrain characteristics, particularly the LS factor and vegetation cover, with higher concentrations observed in smooth and moderately undulating terrain. These findings highlight the importance of monitoring plastic debris in the microwatershed terrain and identifying pollution sources to provide valuable insights for mitigating its environmental impact.

## 1 Introduction

Plastic products are extensively used in the agricultural sector due to their advantageous properties, such as durability, versatility, mechanical resistance, and low cost.^1−3^ These materials serve multiple purposes, including use as cover films, fertilizer and seed bags, irrigation pipes, pesticide containers, and protective netting, among others.^4−6^ While the utility of plastics in agriculture is undeniable, the increasing improper disposal of plastic products has led to significant socio-environmental impacts, including contamination that affects the food chain^7,8^ and degrades soil quality.^9^

Plastic pollution, resulting from waste of various sizes, has been extensively studied due to its significant impact on ecosystems.^8−10^ Once released into the environment, plastic debris is subjected to multiple degradation processes driven by factors such as moisture, oxygen, heat, radiation, light, and mechanical stress, leading to its progressive fragmentation into smaller particles.^11,12^ Research confirms the presence of plastic particles in various ecosystems.^13,14^ It has also been shown that the contaminating potential of these particles in biotic and abiotic environments is directly related to their size.^15,16^ For example, studies have shown that macroplastics in agricultural soils can reduce their bulk density, reduce the number of macropores, and alter the distribution of water in the soil. On the other hand, microplastics can affect the growth and survival rate of soil organisms, thus altering soil functionality.^17^

Plastic debris is classified by size into macroplastics (MaP, >25 mm), mesoplastics (MeP, 5–25 mm), microplastics (MP, 1 μm–5 mm), nanoplastics (NPs, <1 μm, typically 1–1000 nm),^17,18^ and coarse microplastics (GMP, 2–5 mm), a subclass of MP.^19−21^ The fragmentation and degradation of plastics are influenced by factors including polymer type, debris thickness, and soil organisms.^17,26^ Common agricultural polymers include polyethylene (PE), low-density polyethylene (LDPE), high-density polyethylene (HDPE), polypropylene (PP), polystyrene (PS), and poly(vinyl chloride) (PVC). These polymers, composed of carbon–carbon chains, degrade through chain cleavage processes, with degradation times varying by polymer type.^14,27^

Plastic waste in agricultural soils comes from plastic mulch, input packaging, pesticides, organic fertilizers, rural household waste, sludge landfill, composting, wastewater irrigation, road runoff, and atmospheric deposition.^11,22,23^ The accumulation of such debris poses a growing threat to agroecosystems, with the degradation of macroplastics being a primary source of microplastics.^6−8,24,25^

Studies conducted worldwide have explored the abundance, distribution, polymer composition, shape, size, and migration of plastic debris in agricultural soils.^26,28−31^ On the other hand, the accumulation of plastic particles in agricultural soils depends on several factors, including soil type and use, vegetation cover,^32^ and climatic factors associated with its geographical location. Considering the geographical location, plastic debris could easily migrate to surrounding ecosystems, reaching seas and oceans, or traveling along large river basins, causing far-reaching impacts. According to Cai et al.^33^ humid tropical agricultural soils in China exhibited higher plastic debris accumulation due to the influence of regional climate.^9,11,33^ Climatic factors such as temperature and altitude also affect plastic debris accumulation in agricultural soils.^33^

Despite existing studies, several factors linking agricultural soils to plastic debris remain unexplored, including the role of vegetation as a plastic sink, the influence of soil preparation and management, and the dynamics of particle size in agricultural soils.

This study aims to quantify and characterize the abundance, mass, and polymer composition of plastic debris in tropical agricultural soils, particularly in identifying the types and sources of contamination. Additionally, the study aims to analyze the spatial distribution of plastic debris and relate its characteristics (quantity, mass, area, diameter, and perimeter) to the topographic features of the terrain, such as slope, LS factor, and vegetation cover, to identify patterns and potential critical accumulation points. It is important to emphasize that the study area exhibits characteristics similar to those of other regions of Brazil and can serve as a reference for designing effective strategies for plastic pollution prevention and control, promoting sustainable agricultural development.

This study provides a detailed investigation of the characteristics, abundance, types, and spatial distribution of plastic debris (MaP, MeP, and GMP) in agricultural soils within a representative river basin in southeastern Minas Gerais. The study area encompasses forests, grasslands, and agricultural lands where activities, such as soil preparation, fertilization, irrigation, and harvesting, take place. These activities serve as a basis for understanding plastic waste pollution in tropical soils.

## 2 Materials and Methods

### 2.1 Site Description

**Figure 1:**
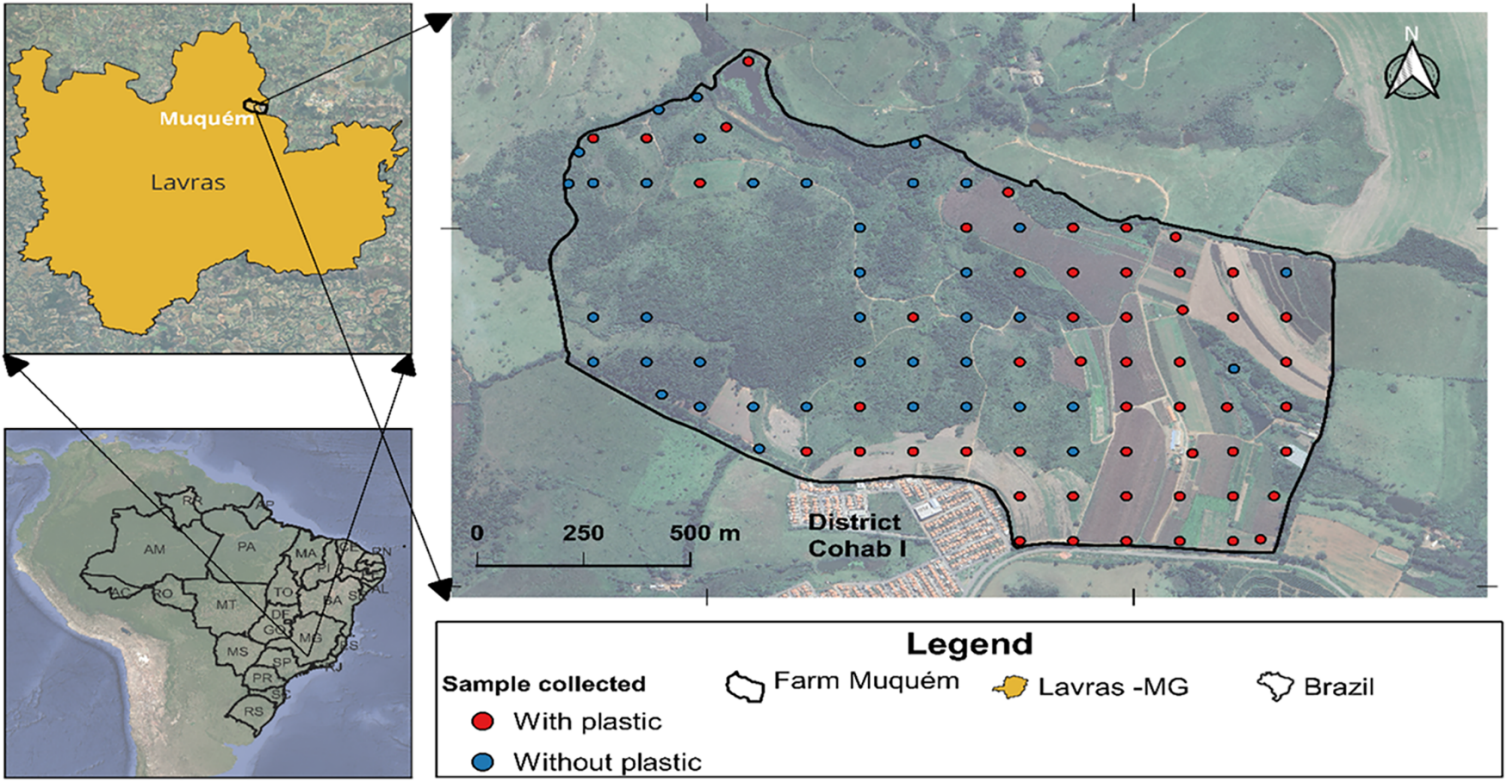
Map of the study area and geographic location of sampled points with and without plastic debris. The base map was obtained from the Google Earth Engine plugin in QGIS software. Map data are 2024 Google.

The study area was the Muquém Experimental Farm of the Federal University of Lavras (UFLA), located in a sub-basin of the Rio Grande in southeastern Brazil. The farm covers an area of approximately 161 ha of diversified land including forest, grassland, and agriculture (Figure. 1). The sub-basin is part of the Atlantic Plateau geomorphological unit, specifically on the surface of the Upper Rio Grande, a tributary of the Paraná River, located in the municipality of Lavras, Minas Gerais. It is between latitude −21°12′037″S and longitude −44°59′3.77″W, WGS 84 UTM 23S.

The climate of the region is classified as Cwa, temperate rainy (mesothermal) according to the Köppen climate system, with a dry winter and subtropical rainy summer, with an average annual rainfall of 1383.4 mm, concentrated in the summer, from October to February.^34,35^ According to the Meteorological Service in Brazil (INMET) database, the prevailing winds are easterly from March to September and northeasterly from November to February,^36^ with average wind speeds of 2.16 m s^–1^ and 1.64 m s^–1^, respectively.

According to the international classification,^37^ the soil classes present in the study area correspond to Inceptisols (CX), which occupy 18.84 ha (11.41% of the area), Entisols Aquepts (GM), which occupy 1.92 ha (1.16% of the area). Oxisols (LVA) occupy 63.60 ha (38.50%), Alfisols (NX) 8.30 ha (5.02%), Ultisols (PVA) 58.07 ha (35.15% of the area), and Lithic Orthents (RL) 14.48 ha, representing 8.76% of the total area of the farm.

**Figure 2:**
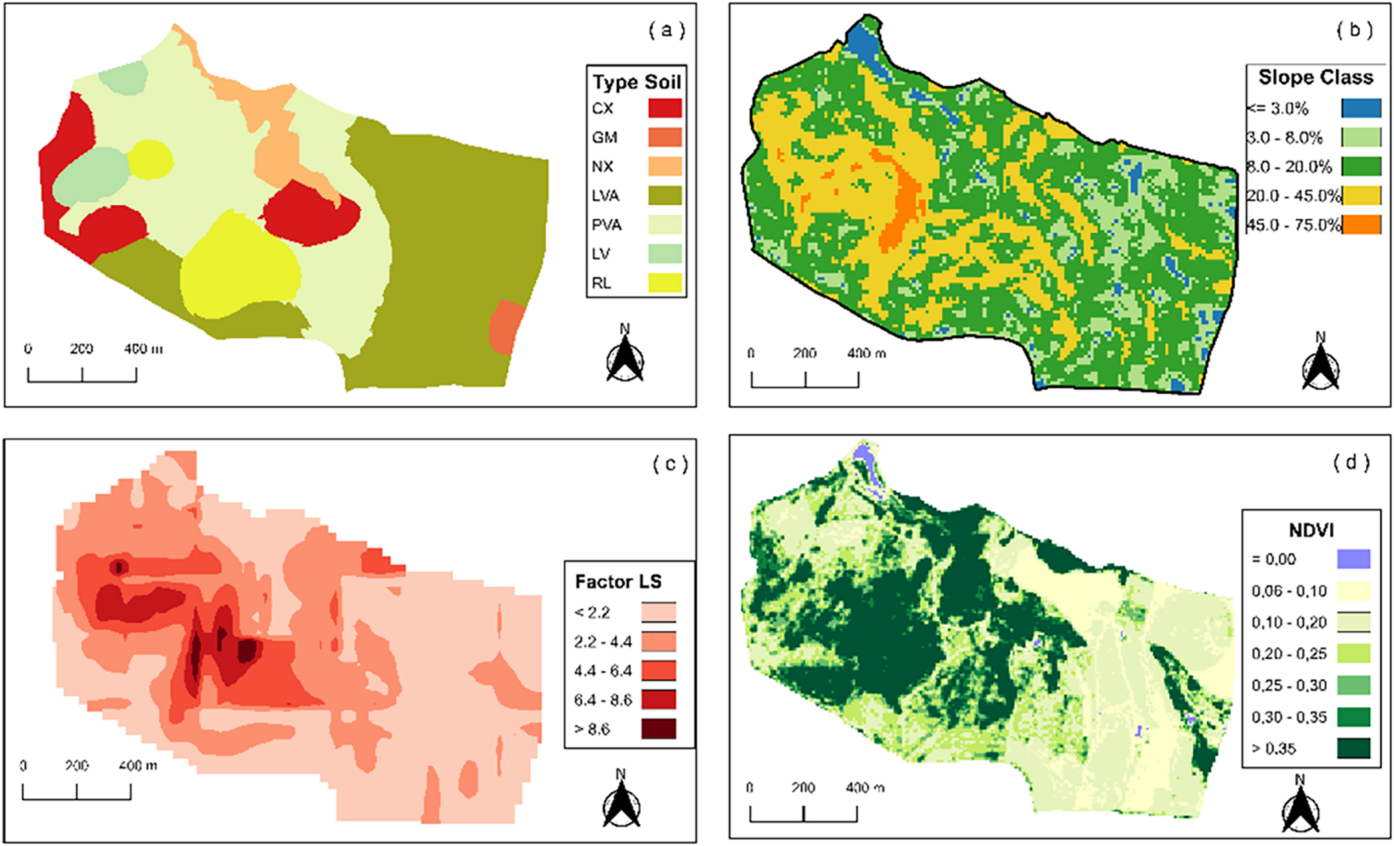
Map showing soil types (a), slope classes (b), LS factor map (c), and NDVI map (d) of the Muquém farm. Note: reliefs associated with their respective slopes, namely: flat (<3%), slightly undulating (3–8%), moderately undulating (8–20%), undulating (20–45%), and highly undulating (45–75%). To Oxisols (LVA), Inceptisols (CX), Ultisols (PVA), Alfisols (NX), Lithic Orthents (RL), and Entisols (GM).

The sub-basin has a minimum elevation of 877 m and a maximum of 1040 m, with varying landforms and slopes. Its landforms are classified according to Silva et al.^37^ as flat (<3%), gently undulating (3–8%), moderately undulating (8–20%), undulating (20–45%), strongly undulating (45–75%), corresponding to 2.7, 17.8, 58.8, 20.1, and 0.6% of the total area of the farm, respectively, as seen in Figure 2b.

Land use on the farm was classified into three types.^37^ The first is agricultural use, with annual crops representing 29.15% of the agricultural land, with maize (*Zea mays*), soybean (*Glycine max* (L.) *Merrill*), wheat (*Triticum aestivum* L.), beans (*Phaseolus vulgaris*), sorghum (*Sorghum bicolor* L.), and rice (*Oriza sativa* L.), as well as perennial crops (0.91% of the area), coffee (*Coffea arabica*), eucalyptus (*Eucalyptus* sp.), and, to a lesser extent, other forest species. This is followed by grassland (mainly Poaceae species), with 39.33% cultivated grassland and 4.69% native grassland, and then forest, with 24.39% of the farm area.

### 2.2 Sampling of Study Material

Plastic debris was collected between August 2022 and April 2023. First, the study area was surveyed, and sampling points were georeferenced. The surrounding vegetation, land use, and altitude were determined. Georeferencing was carried out using a Garmin Oregon 750 Global Positioning System (GPS) with an accuracy of 3 m, using a regular grid of 125 × 125 m^2^ resolution. A total of 91 points were georeferenced, corresponding to a sampling density of 1.77 samples per hectare.

At each sampling point, the soil surface was visually inspected for plastic debris, which was collected within an area of approximately 19.63 m^2^ per point. The samples were collected, stored, identified by mesh point, and transported to the laboratory for further analysis.

To determine the topographic characteristics of the study area, such as the topographic factor (LS factor) and the normalized difference vegetation index (NDVI), secondary geomorphometric information from the TOPODATA database of the National Space Research Institute (INPE) and maps from the CBERS 4 satellite in October 2022 were used.

The LS factor was determined using the tool SAGA-GIS, “LS-Factor, Field-Based”, following the method of Desmet and Govers.^38^ The NDVI and LS factor values were extracted using the Point Sampling Tool plugin available in the QGis software.^39^Figure 2c,d shows the map of the LS factor and the vegetation index (NDVI).

### 2.3 Classification, Characterization, and Identification of Plastic Debris

The plastic debris collected at each sampling point was classified according to its mass, dimensions (length, perimeter, and area), and polymer type. The classification of plastic debris was also carried out considering land use. To follow, the collected plastic particles were first physically shaken and washed with distilled water to remove the remnants of soil and nonplastic materials adhering to their surface. They were then air-dried and stored in steel containers.

Each piece of plastic debris was then photographed by using a high-resolution digital camera (Canon EOS 60D). The digital images obtained were analyzed using ImageJ software.^40^ The photographs of the debris, together with the scale of a caliper (±0.5 mm), helped to determine the pixel of the digital image and hence the dimensions of the debris. Based on these measurements, plastic debris was classified as macroplastics (MaP) for particles >25,000 μm, mesoplastics (MeP) for particles between 5000 and 25,000 μm, and coarse microplastics (GMP) for particles between 2000 and 5000 μm.

To calculate the mass and abundance of plastic debris per sampling point, eqs 1 and 2 were used, respectively. These values were then extrapolated to hectares using a conversion factor (*f*_1_)
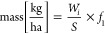
1
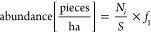
2where *W*_i_ (g) is the total mass of plastic debris, *S* (m^2^) is the surface area of the sampling point (approximately 19.63 m^2^), *N*_i_ is the number of plastic particles per sampling point, and *f*_1_ is the conversion factor to hectare. The data from eqs 1 and 2 were recorded in a georeferenced database using QGIS software.

Raman spectroscopy was used to identify the type of polymer present in the plastic debris collected at each point. Raman spectra were obtained at room temperature by using a confocal Raman microscope (Alpha300 from Witec, Germany) with a 785 nm excitation laser and a 50× magnification objective. Depending on the sample analyzed, the laser power varied from 2 to 15 W, the integration time from 1.0 to 2.0 s, and the accumulations from 20 to 30 cycles. All measurements were performed in the spectral range 300–3500 cm^–1^. The Raman spectra were plotted using OriginPro 2024b software.^41^ Raman spectroscopy analysis was conducted by comparing the samples’ Raman spectra with the Raman spectra of polymers of reference obtained from the literature. Thus, the march in the positions and intensities of the bands between the sample and reference spectra enabled the identification of the polymer type with high confidence. From this analysis, the identified Raman spectra corresponded to polypropylene (PP), polyethylene (PE), poly(vinyl chloride) (PVC), poly(ethylene terephthalate) (PET), polystyrene (PS), polyamide (PA), rubber, and acrylic.

### 2.4 Statistical Data Analysis

The data analysis aimed to test whether there was a difference in plastic debris’s abundance, mass, and geometric dimensions depending on the land use in the study area. To do this, the distributions of the groups were compared using the Kruskal–Wallis test, which is suitable for data that do not follow a normal distribution. Dunn’s test was then used to make multiple comparisons between group medians. Results were considered statistically significant if the *P* value was less than 0.05. Point distribution maps of the abundance (pieces ha^–1^) and mass (kg ha^–1^) of plastic debris per sampling site were generated using QGIS software (Development Team 2024).

In addition, a multiple factor analysis (MFA) was performed,^42,43^ which explores the relationship between the topography variables (slope and LS factor) and the NDVI index with the abundance and size variables of plastic debris collected on the soil surface. Three databases corresponding to four slope bands, seven LS factor bands, and seven NDVI index bands were constructed to perform the MFA (Tables S1–S3).

In each table band, the average of two groups was recorded: the first called “abundance”, which includes the quantity and mass of plastic debris; and the second called “dimensions”, made up of the area, diameter, and perimeter of the plastic debris. Statistical analysis and MFA in software R^44^ using the MVar.pt library.^43^

## 3 Results

### 3.1 Abundance and Point Distribution of Plastic Debris in the Study Area

**Figure 3:**
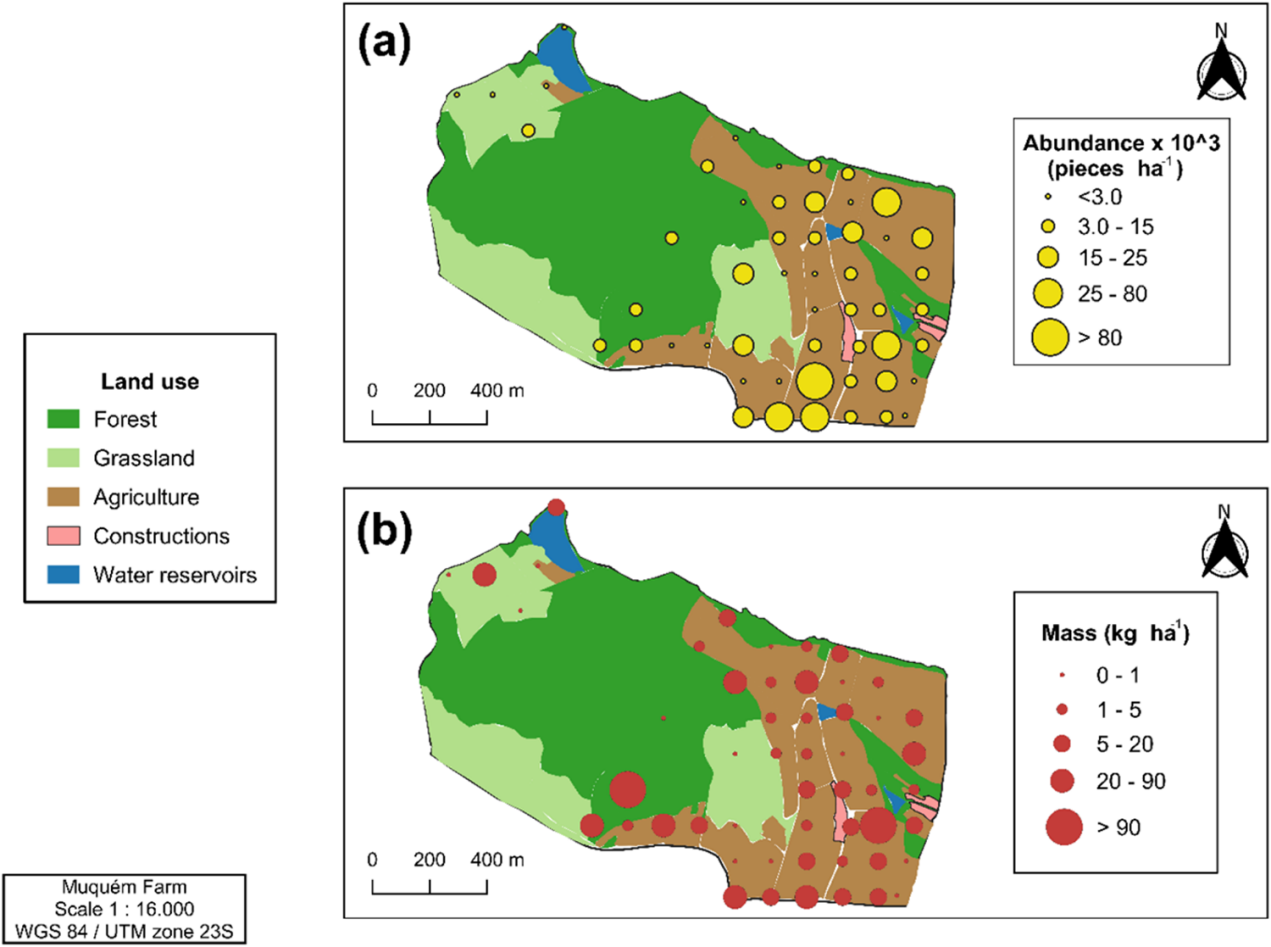
Map of the studio area showing the spatial distribution of the abundance (a) and mass (b) of plastic debris associated with land use.

The spatial distribution of plastic debris abundance (Figure 3a) and mass (Figure 3b) in the study area was analyzed based on land use. Out of 91 georeferenced sampling points on Muquem Farm, plastic debris was found at 56.52% of the points, with 44.56% in agricultural areas, 6.52% in forested areas, and 5.44% in grasslands. Conversely, no plastic debris was detected at 43.48% of the points, distributed as follows: 22.83% in forested areas, 15.22% in grasslands, and 5.43% in agricultural land. These results highlight the predominance of plastic debris in agricultural zones.

The abundance of plastic debris, estimated per soil area, ranged from 0.509 × 10^3^ to 104 × 10^3^ pieces ha^–1^. In the distribution map (Figure 3a), the litter abundance was described according to the model proposed by Liu et al.^45^ and Weber et al.^46^ Very high represents abundance values greater than 80 × 10^3^ pieces ha^–1^, high represents values between 25 × 10^3^ and 80 × 10^3^ pieces ha^–1^, medium represents values between 15 × 10^3^ and 25 × 10^3^ pieces ha^–1^, low represents values between 3 × 10^3^ and 15 × 10^3^ pieces ha^–1^, and very low represents values less than 3 × 10^3^ pieces ha^–1^.

The map of plastic debris mass distribution by soil area followed the same logic as described above. Where the category very high represents mass values greater than 90 kg ha^–1^, high represents values between 20 and 90 kg ha^–1^, medium represents values between 5 and 20 kg ha^–1^, low represents values between 1 and 5 kg ha^–1^, and very low represents values less than 1 kg ha^–1^.

**Figure 4:**
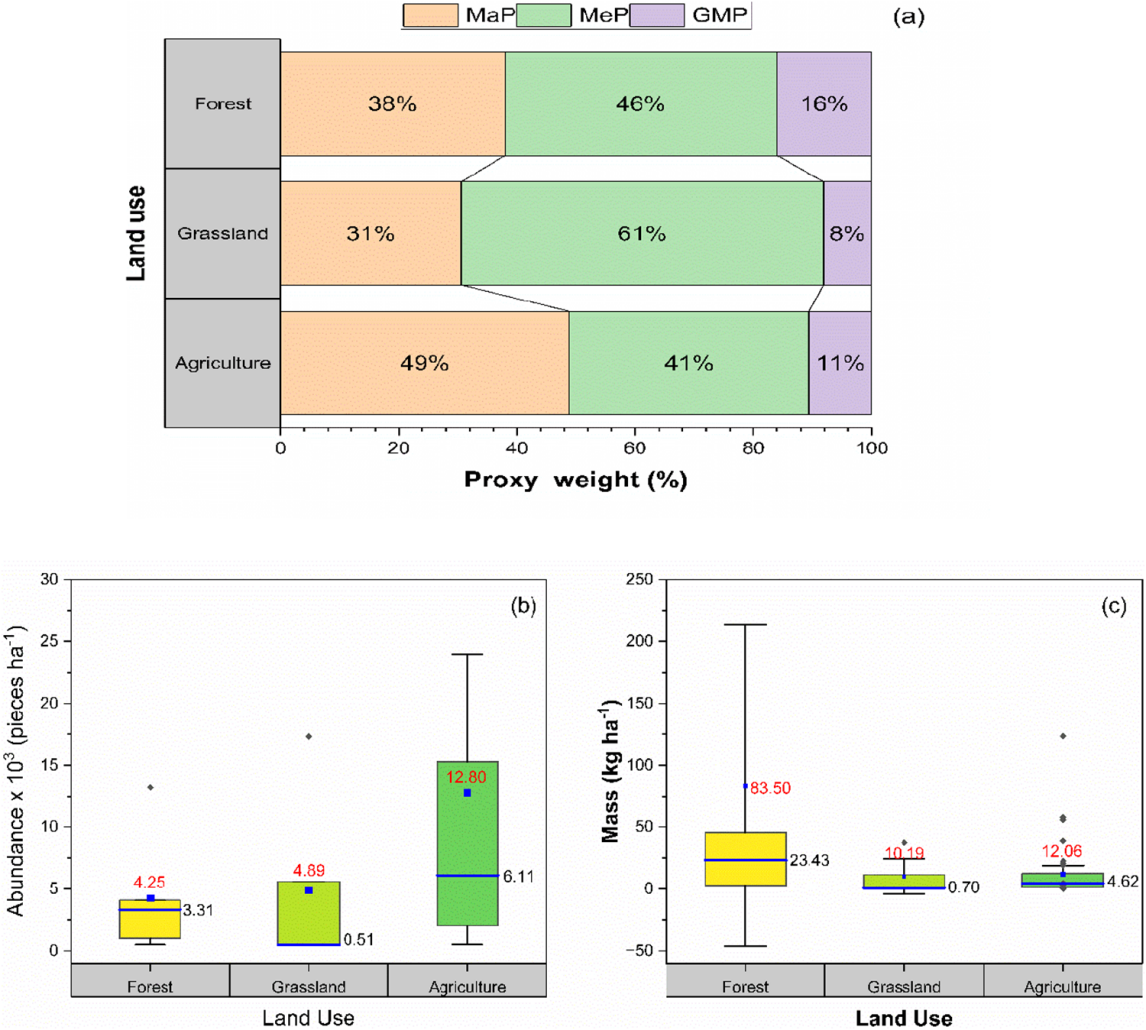
Cumulative percentage abundance of macroplastics (MaP), mesoplastics (MeP), and coarse microplastics (GMP) (a). Boxplot of abundance (b) and mass (c) of plastic debris collected about land use. The diagrams show extreme values (whiskers), the first and third quartiles (the bottom and top of the boxes, respectively), and the median value (the line inside the boxes with the black number next to the line). The average of the data is represented by the red numbers in red.

The cumulative abundance of plastic debris was dependent on the sites sampled (Figure 4a). The MaP and MeP plastic debris size classes were the most representative of the three land uses studied. When analyzing the total plastic debris by size, it was observed that the agricultural area had the highest average percentage of MaP, while the grassland and forest areas had the highest percentage of MeP. On the other hand, GMP was found in smaller quantities in all sampled areas compared to debris of other sizes (Figure 4a).

The mean abundance of plastic debris (Figure 4b) was 4.25 × 10^3^, 4.89 × 10^3^, and 12.80 × 10^3^ pieces ha^–1^ observed for the forest, grassland, and agriculture areas, respectively.

The spatial distribution of the mass of plastic debris on the soil surface at each sampled site varied from 0.039 to 405 kg ha^–1^ (Figure 3b). The total average as a function of land use was 83.60 kg ha^–1^ for forest, 10.18 kg ha^–1^ for grassland, and 12.05 kg ha^–1^ for agriculture (Figure 4c). There were no significant differences between the mean abundance and mass of plastic detritus for the three investigated land uses (forest, grassland, and agricultural land).

### 3.2 Dimensions of Plastic Debris in the Agricultural Sub-Basin

A total of 1123 pieces of plastic debris were collected from 52 points, representing a total abundance of 572.08 × 10^3^ pieces ha^–1^. Table 1 shows the abundance of plastic debris classified by land use and debris size. The results show that 4.45% of the plastic debris was found in the forest area, 4.36% in the grassland area, and 91.2% in the agricultural area.

**Table 1:** Land Use, Size Class of Plastic Debris (*S*), Total Abundance (*n*), and Geometric Dimensions of Plastic Debris^a^^b^

				area (cm^2^)		length (cm)		perimeter (cm)	
land use	S	*n* × 10^3^ (pieces ha^–1^)	FQR (%)	median	iqr	median	iqr	median	iqr
forest	MaP	9.68	1.69	42.991	354.890	15.676	30.594	67.252	91.708
	MeP	11.72	2.05	0.251	0.531	0.778	0.557	2.215	2.191
	GMP	4.08	0.71	0.084	0.025	0.441	0.082	1.316	0.357
grassland	MaP	7.64	1.34	15.570	42.286	7.360	5.916	28.580	26.095
	MeP	15.28	2.67	0.210	0.160	0.735*	0.528	2.520	1.178
	GMP	2.04	0.36	0.043	0.067	0.328	0.234	1.115	0.953
agriculture	MaP	255.22	44.6	16.170	40.790	8.280	9.655	32.610	38.786
	MeP	211.41	36.5	0.210	0.325	1.090*	0.797	3.160	2.695
	GMP	55.02	9.62	0.030	0.030	0.340	0.150	0.865	0.392

a Median and interquartile range (IQR).

b Note: Macroplastic (MaP), mesoplastic (MeP), coarse microplastic (GMP). *n*: Total number of plastic debris by land use and size class (pieces ha^-1^). FRQ: fraction corresponding to the size of plastic debris in a given range out of the total number (1123 samples) * Significant differences by Dum test at significance level *P* < 0.05.

This indicates that the agricultural area has the highest concentration of debris on the soil surface. Specifically, in the agriculture area, MaP represented 44.6%, MeP 37.0%, and GMP 9.6% of the debris. In the grassland area, MaP, MeP, and GMP made up 1.3, 2.7, and 0.4% of the debris, respectively. In the forest area, these values were 1.7% for MaP, 2.0% for MeP, and 0.7% for GMP (Table 1).

The Kruskal–Wallis test showed no significant differences between the area and perimeter of plastic litter in the three land uses. However, a significant difference was observed when the litter length values were compared according to the land use sampled (Table 1). The results showed that the plastic debris with the smallest area, length, and perimeter was found in the agricultural area, with the smallest values recorded being 0.01 cm^2^, 0.20 cm, and 0.42 cm, respectively. On the other hand, the plastic debris with the largest area and length was found in the forest area, with maximum values of 5076 cm^2^ and 94 cm, respectively.

**Figure 5:**
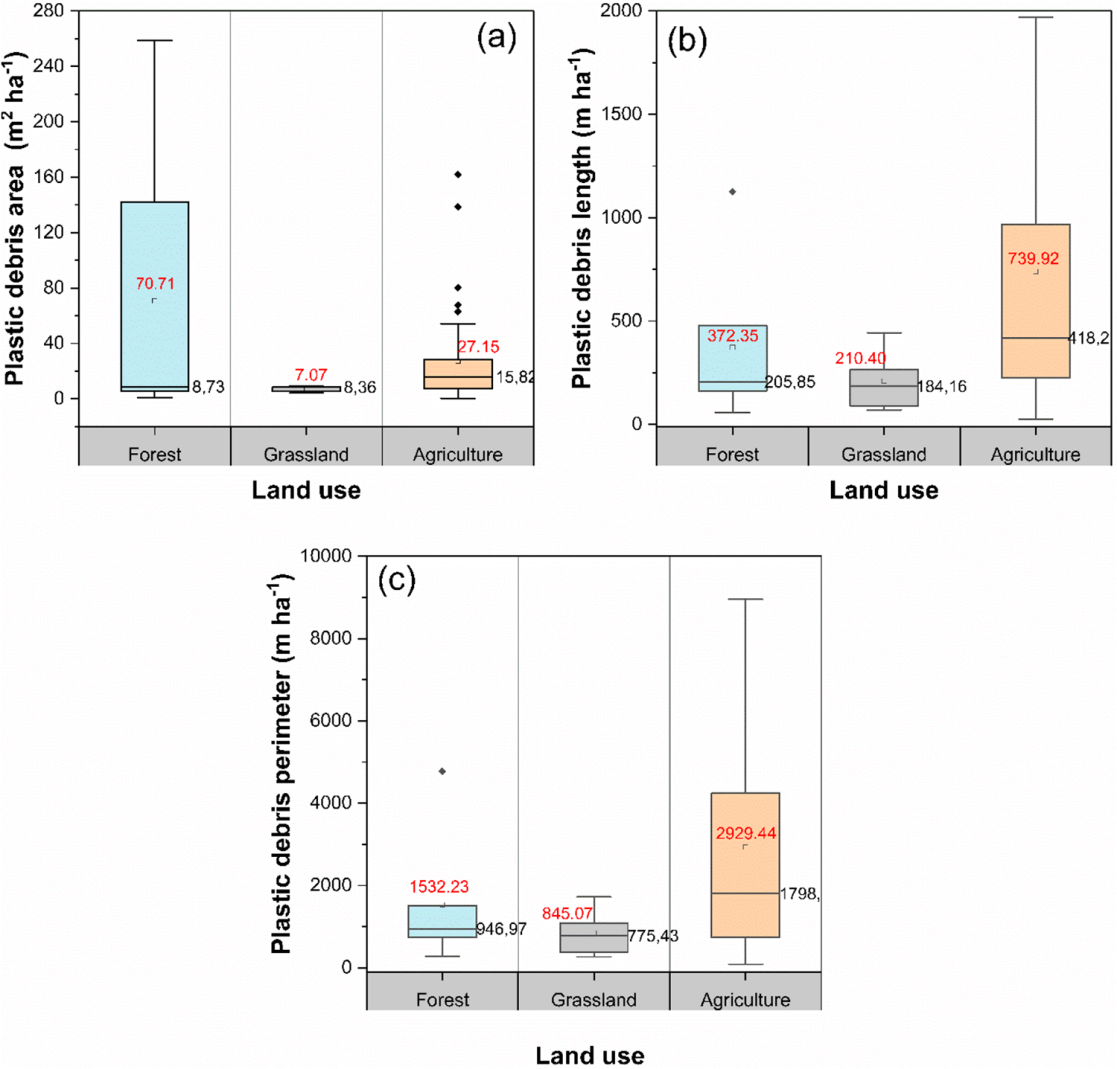
Box plots of the distribution of the area (a), perimeter (b), and length (c) by land use on the Muquém farm. The plots show the extreme values (whiskers), the first and third quartiles (the bottom and top of the boxes, respectively), and the median (the line inside the boxes with the black number next to the line). The average of the data is represented by the numbers in red.

Figure 5 presents the extrapolated dimensions of plastic debris (area, perimeter, and length) for each land use on the Muquem farm, standardized to 1 ha. Statistical analysis revealed no significant differences in the dimensions of plastic debris among the three land uses. Plastic debris collected from agricultural land exhibited the largest interquartile ranges for length (225.31 to 966.94 m ha^–1^) and perimeter (734.38 to 4242.44 m ha^–1^). In contrast, forest areas showed the widest interquartile range for the area of plastic debris, varying between 5.89 and 108.97 m^2^ ha^–1^. Agricultural land had the highest mean values for all dimensions, with an average area of 27.15 m^2^ ha^–1^, length of 739.92 m ha^–1^, and perimeter of 2929.43 m ha^–1^.

### 3.3 Relationship between the Topographic Conditions and the Vegetation Index (NDVI) of the Study Area and the Abundance and Size of the Plastic Debris

The quantity, mass, area, diameter, and perimeter of plastic debris varied with land topography, specifically slope class, LS factor, and NDVI vegetation index. In forest areas, plastic debris was found only in moderately undulating relief, despite covering all relief types. Grassland areas, dominated by moderately undulating relief, showed the highest level of plastic debris accumulation. Agricultural areas contained plastic debris across all relief types except undulating and strongly undulating, which were scarce (Figure 2b).

Overall, 2% of debris was found in undulating terrain, 65% in moderately undulating terrain, 25% in gently undulating terrain, and 8% in flat terrain. The LS factor ranged from 1.711 to 4.967 in forests, 2.123 to 3.160 in grasslands, and 0.126 to 2.748 in farmlands. Agricultural areas showed the highest debris presence and the lowest LS factor, particularly in the 0.126–2.201 range.

NDVI values ranged from 0.071 to 0.354 in forests, 0.119 to 0.229 in grasslands, and 0.060 to 0.380 in farmlands. Notably, 61% of agricultural sampling points fell within the NDVI range of 0.060–0.152 (Figure 2d).

**Figure 6:**
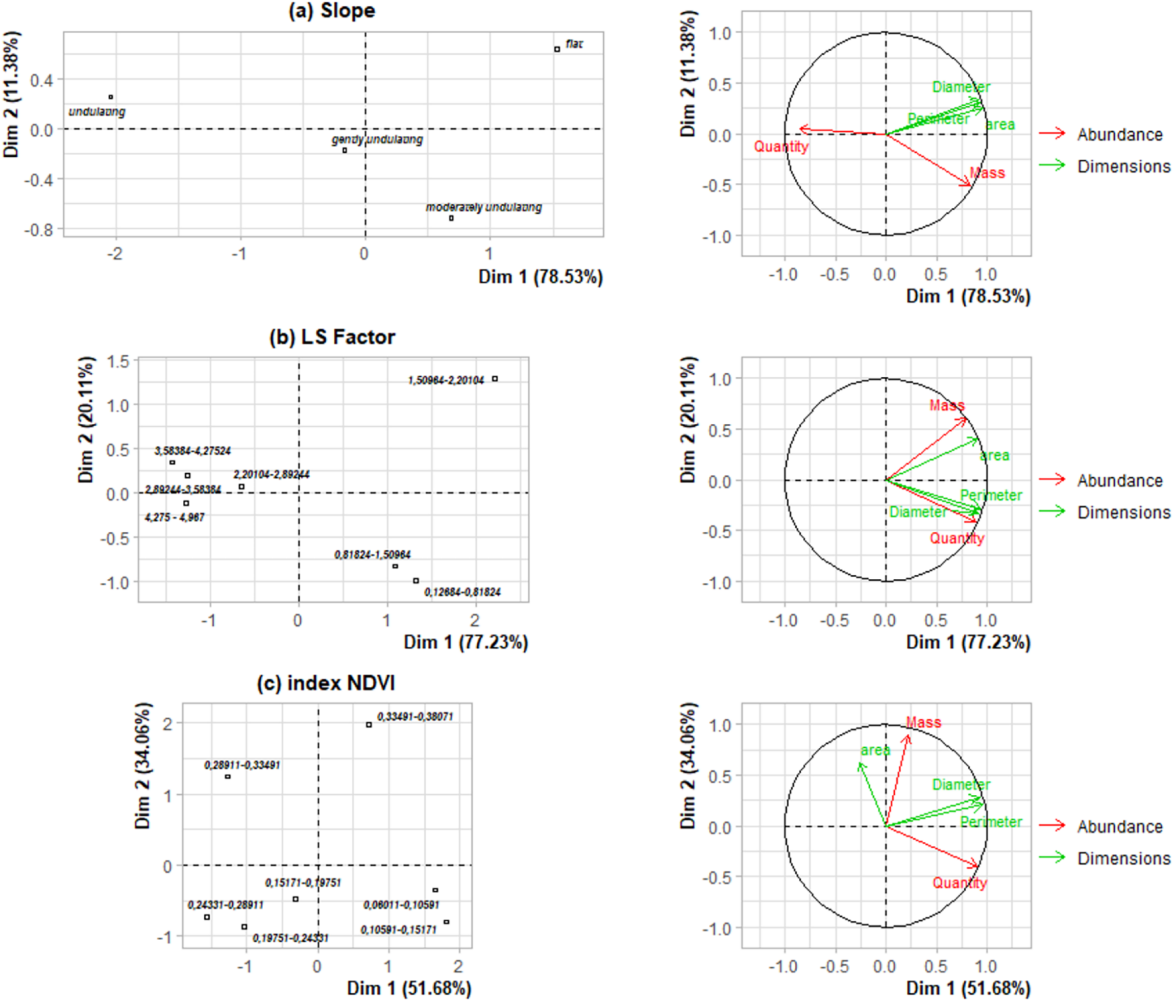
Multiple factor analysis (MFA) of the variables (a) slope, (b) LS factor, and (c) NDVI index as a function of plastic debris abundance and size groups.

The exploration analysis of these variables was performed using multiple factor analysis (MFA) (Figure 6), which includes three databases, with slope, LS factor, and NDVI index being the individuals for each database (Tables S1–S3, respectively). Five variables are grouped into two groups: the abundance group contains the variables quantity and mass, and the dimensions group contains the variables area, diameter, and perimeter of plastic debris.

For the MFA of the slope variable and groups (Figure 6a), two components were derived that are responsible for explaining 89.91% of the total variation. The first component shows a variability of 78.53%, with the variables in the dimension group consisting of area, diameter, and perimeter having coefficients of 0.956, 0.921, and 0.930, respectively. The second component showed a variability of 11.38%. This variability was mainly explained by the plastic debris mass variable with a coefficient of −0.509.

For the MFA of the LS factor variable and the groups (Figure 6b), two components were derived that are responsible for explaining 97.34% of the total variation. The first component shows a variability of 77.23%, with the variables in the dimension group consisting of area, diameter, and perimeter having coefficients of 0.907, 0.941, and 0.921, respectively. The second component showed a variability of 20.11% explained by the variables in the abundance group, made up of the quantity and mass of plastic debris, with coefficients of −0.411 and 0.608, respectively.

For the MFA of the NDVI index variable and the groups (Figure 6c), two components were derived that are responsible for explaining 85.74% of the total variation. The first component shows a variability of 51.68%, where the variables perimeter, diameter, and quantity have coefficients of 0.955, 0.923, and 0.907, respectively. The second component showed a variability of 34.06% explained by the variables mass and area of plastic debris, with coefficients of 0.902 and 0.625, respectively.

### 3.4 Spectral Composition of the Polymers

Raman spectrometry was used to fingerprint each material and identify the type of polymer in the debris that was analyzed. From the total of 1123 pieces of plastic debris collected at the 52 sampling points, 191 groups with very similar characteristics were formed. Representative samples were selected from each group based on the characteristics of the debris, such as size, shape, and color. In addition, the history of use in the sampled areas was taken into account, such as irrigation with water containing plastic, common litter such as plastic bags, debris from roads or nearby communities, and others.

Spectra were collected from different regions of the surface of each sample analyzed. The Raman spectra obtained showed baseline deviation and varying signal intensities; therefore, corrections were made, including noise removal, baseline correction, and normalization.

**Figure 7:**
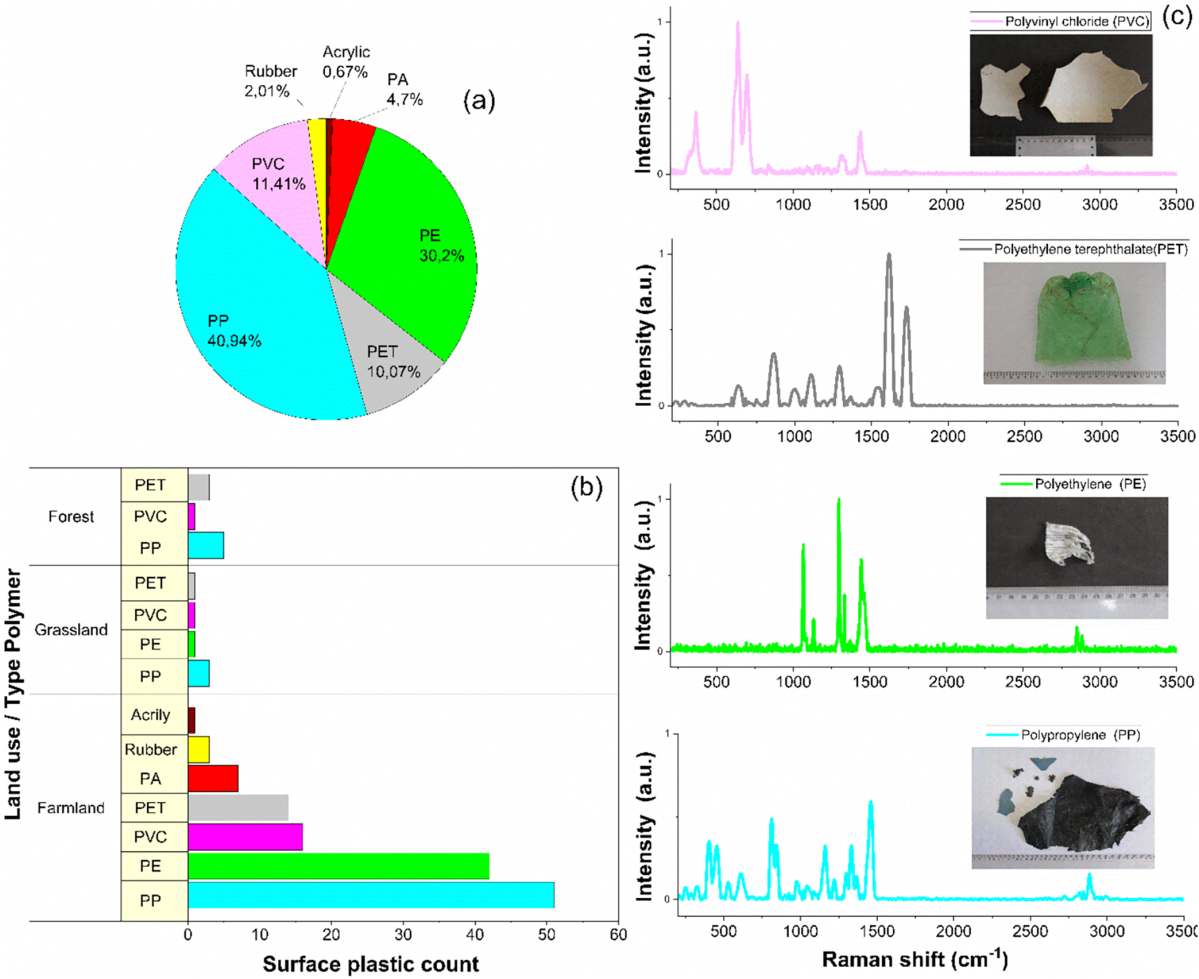
Polymer type and percentage distribution (a), number of plastic debris by polymer type and by land use (*n* = 191) (b), and spectrum of polymers by Raman spectroscopy (c), collected on the soil surface of the study area. PE, polyethylene; PP, polypropylene; PVC, poly(vinyl chloride); PET, poly(ethylene terephthalate); PS, polystyrene; PA, polyamide..

The results indicated that the primary polymer types identified among the samples analyzed were PP, PE, PVC, PET, and PA. As shown in Figure 7a, PP and PE were the most prevalent, accounting for 40.94 and 30.2% of the total polymers, respectively. PVC, PET, and PA constituted 11.4, 10.1, and 4.7%, respectively, while other polymers, including rubber and acrylic, represented 2.68% of the total. The Raman spectra (Figure 7c) of the most representative polymers found in the samples analyzed were those of PP, PE, PVC, and PET. In all cases, the spectra showed characteristic peaks for each type of polymer.

The Raman spectra corresponding to PP showed a characteristic peak at around 841 cm^–1^, representing the vibrations of its helical molecules. The peak at 1330 cm^–1^ is associated with the vibration of PP macromolecules in the helical conformation, corresponding to a superposition of the deformation mode of the C–H bond and the torsion mode of the CH_2_ groups. The peak at 1435 cm^–1^ can be attributed to the asymmetric bending mode of the −CH_3_ group, while the peaks at 1252 and 1170 cm^–1^ are due to the stretching of the C–C structure.

In the Raman spectra of the samples identified as PE, characteristic peaks were observed in the 1000–1500 and 2800–2900 cm^–1^ regions. The peaks with the highest intensity were located at 1066, 1298, and 1439 cm^–1^. These peaks are attributed to C–C stretching, CH_2_ torsion, and CH_2_ symmetric deformation, respectively. In addition, a peak at 1132 cm^–1^ corresponding to C–C stretching was observed.

The PVC samples showed spectra with high-intensity Raman peaks at 640 and 698 cm^–1^ attributed to the stretching vibrations of C–Cl bonds. The peak at 1435 cm^–1^ corresponds to the angular deformation of the CH_2_ and CH_3_ groups. Finally, the PET spectrum showed two dominant peaks located at 1615 and 1729 cm^–1^. These correspond to the C–C stretching vibrations of the aromatic ring and the stretching vibrations of the carbonyl groups.

The analysis of polymer identification by Raman spectroscopy revealed that polypropylene (PP) is the main source of plastic pollution in the sampled areas, regardless of the type of land use (Figure 7b). In addition, it was observed that agricultural soils accumulate a greater variety of polymer types compared with the other areas.

## 4 Discussion

### 4.1 Land Use-Related Plastic Debris

This study revealed the presence of plastic debris of varying sizes and chemical compositions on the soil surface of a subwatershed in southeastern Brazil. Most of the debris was concentrated in agricultural areas, while forest and grassland regions showed lower contamination levels. This pattern highlights the strong influence of anthropogenic activities on the accumulation of plastic waste, particularly agriculture activities. A similar situation was described by Parolini et al.^47^ Furthermore, it has been reported that low-density polyethylene (LDPE) and polypropylene (PP) are commonly used in agricultural materials, such as greenhouse covers, nets, and films.^24^ Consistently, our study confirmed that PP and polyethylene (PE) prevailed at the study site, comprising 40.9 and 30.2% of the total debris analyzed, respectively (Figure 6a) as confirmed by Raman spectroscopy.

As previously mentioned, the Raman spectra of the analyzed plastic debris matched the known polymer spectra in the reference library. These results are consistent with Raman analyses of plastic debris found in soils across different regions.^48−50^ However, the spectra of some samples exhibited bands that shifted toward lower wavenumbers compared with the reference spectra, as observed in the PP spectrum (Figure 7c). This result suggests that weathered plastics undergo structural changes related to variations in molecular bond lengths, as indicated by Phan et al.^48^

Wang et al.^16^ reported an abundance of 266.2 × 10^4^ ± 507.9 pieces ha^–1^ of PE plastic waste used as mulch film in agricultural soils in northwest China. In southeastern Germany, 56% of the plastic debris found in agricultural soils was identified as PE polymers, with an average abundance of 206 pieces ha^–1^. In agricultural soils in central Germany, the predominant polymers detected were PS, PP, and PE, with a total abundance of 637 pieces ha^–1^.^46^ These findings align with Kawecki and Nowack’s^7^ study, which highlighted similar trends in macroplastic and microplastic emissions in agricultural regions of China.

At our experimental site, the average abundance of plastic debris was 12.79 × 103 pieces ha^–1^ in the agricultural area, 4.89 × 10^3^ pieces ha^–1^ in the grassland area, and 4.24 × 10^3^ pieces ha^–1^ in the forest area (Figure 4b). These results show variations in the abundance of plastic debris compared to that in other studies. Such differences can be attributed to several factors, one of which is the management of plastic materials in soils under agricultural use.^51^

The results showed that the highest mass concentration of plastic debris was recorded in the forest area, averaging 83.50 kg·ha^–1^ (Figure 4). This result was expected, since no anthropogenic activities in this region significantly affected the size of the debris. In contrast, agricultural land exhibited a higher abundance of MaP, MeP, and GMP particles (Table 1), consistent with Meng et al.,^17^ who reported that intensive tillage practices accelerate plastic fragmentation, increasing microplastic (MP) concentrations compared to other land uses.^52^

The mass of plastic debris in agricultural soil (12.06 kg·ha^–1^) was lower than the values reported in previous studies. For instance, Wang et al.^16^ documented an average of 47.2 kg·ha^–1^ for MaP-sized debris, while Meng et al.^17^ reported 53.7 kg·ha^–1^ in agricultural soils of Northwest China. Berenstein et al.^51^ found a range of 25 to 31 kg·ha^–1^ in Argentinian horticultural soils, and Lero et al.^31^ recorded an average of 60 kg·ha^–1^ of MaP in the topsoil of forest islands formed by the Dunajec River in southern Poland. Despite the lower plastic debris mass in agricultural areas, continuous monitoring and regulatory measures are essential to mitigate plastic pollution in these environments.

We observed that in forest areas, the type and density influence the accumulation of plastic debris. Specifically, the highest NDVI values correspond to points with the highest mass concentration of plastic debris (Figures 2d and 4b). The type and density of vegetation play a key role in retaining plastic debris within these agroecosystems, preventing its mechanical fragmentation and transport. Vegetation acts as a physical barrier, limiting the direct exposure of plastic debris to environmental degradation factors such as UV radiation, wind, water, and erosion.^31,52^

In summary, it is well known that a significant part of the plastic debris in agricultural soils remains buried as mesoplastics for long periods, and another part will fragment into micro- and nanoplastics,^9,47^ further aggravating the contamination of the soil and terrestrial ecosystem. An interesting macroplastic fragmentation process is explained by Berenstein et al.^51^ They explain that the natural cycles of hydration and dehydration of agricultural soils play a critical role in the decomposition of macroplastics into mesoplastics, microplastics, and even nanoplastics. These natural processes act as plastic shredders in the environment, contributing to the formation of particles smaller than 2 mm.^4,7^ Therefore, detecting and identifying plastic debris in agricultural soils are essential to mitigate their growing presence and environmental impact.

### 4.2 Relationship of Plastic Debris to Landforms and Vegetation

Our findings revealed a correlation between the quantity and mass of plastic debris and the topography of the land, particularly in gently to moderately rolling terrains. These areas, which accounted for 76.6% of the total study sites, are predominantly associated with agricultural activities (Figure 6a).

Several studies have explored the relationship among the migration of plastic debris, ground conditions, vegetation, and human activities. Cao et al.^51^ highlight that most plastic debris tends to accumulate at depths of 0–10 cm, where agricultural activities and soil disturbance are more prevalent. In sloping terrain, plastic debris often concentrates on depressions or less steep areas. Similarly, Wang et al.^52^ identify the application of organic fertilizers and sewage sludge on agricultural land as significant sources of plastic debris. Furthermore, areas with moderate slopes experience slower water runoff, facilitating the accumulation of plastic waste.^53^ High concentrations of plastic debris are also strongly linked to regions with intense anthropogenic activities, particularly agricultural zones.^54^

The LS factors ranged between 0.126–0.818 and 0.818–1.509, located in agricultural areas, and were found to be associated with a higher concentration of plastic debris (Figure 6b). On gentle slopes, plastic accumulation may be greater due to the reduction in water flow velocity, which facilitates plastic deposition on the bottom according to Cesarini and Scalici.^30^ These observations are consistent with the results of Wang et al.,^55^ who indicated that the shorter length of slopes and the low gradient contribute to an increase in the abundance of plastic debris.

Concerning the NDVI index, the lowest bands, between 0.060–0.105 and 0.105–0.151, correspond to areas with low vegetation cover (agricultural use), where a greater amount of low-mass plastic debris was found (Figure 6c). However, Cesarini et al.^19^ describe the opposite scenario on sloping land with greater vegetation cover, plastic debris retention may be more efficient, while areas without vegetation and with a slope favor the dispersal of this debris. However, Figure 6c shows that the mass of plastic debris is greater in the higher NDVI bands, between 0.335 and 0.381, which are associated with forest areas, albeit with less debris (Figure 3a,3b). In these areas, plastic fragmentation and degradation are less intense due to less exposure to physical factors and less human disturbance, which explains the presence of debris with greater mass in regions with higher NDVI index.^56^

As mentioned previously, vegetation cover can act as a natural barrier that traps plastic debris, limiting its migration by reducing surface runoff^9^ and promoting localized accumulation. However, the effectiveness of this barrier depends on the density and type of vegetation present.^51^ For example, agricultural areas with homogeneous crops and vacant lots exhibited greater dispersion and abundance of plastics compared to grassland and forest areas, which are characterized by denser and more diverse vegetation and consequently were found to have higher plastic concentrations, as observed in Figures 3b and 4c.

The size of the plastic debris is related to the flat terrain (Figure 6a), values lower than 2.201 for the LS factor (Figure 6b), and the higher percentage of GMP-type plastic debris is more related to forest areas, which can be explained by the influence of tree foliage,^57,58^ which can trap airborne microplastics, as reported by Huang et al.^59^ and Falakdin et al.^60^

Under these premises, it is assumed that the abundance, size variation, accumulation, and directional redistribution of plastic debris in the soil has multiple possibilities of occurrence, such as the characteristics of the terrain, slope, land use, and variables such as wind, surface runoff, wind erosion,^53,61,62^ the density and type of vegetation,^30^ in addition to human activities in agriculture.^60^ Therefore, a deeper understanding of the mechanisms and processes involved in the distribution and accumulation of plastic debris in soils is essential for advancing knowledge in this field.

### 4.3 Limitations, Future Research, and Recommendations

The spatial distribution map of plastic debris (Figure 3) identifies key accumulation zones, providing a valuable tool for guiding the placement of waste collection points. This is particularly relevant for agricultural areas with flat or gently rolling terrain and sparse vegetation, as highlighted by the multiple factor analysis (Figure 6). Implementing targeted collection strategies in these high-concentration areas could significantly reduce plastic waste accumulation and reduce its environmental impact.

A limitation of this study was the smaller number of samples from forest areas and pastures compared to those from agricultural areas. Dense vegetation in forest areas hindered access, while several sampling points in pasture areas contained no plastic debris of the sizes analyzed. Thus, further research in these areas is necessary. Additionally, there is a lack of studies analyzing other factors influencing plastic accumulation, such as wind patterns, surface runoff dynamics, and temporal variations in plastic debris migration and degradation, as highlighted by Billings et al.^63^

## 5 Conclusions

We present an in-depth analysis of the distribution of plastic debris on a medium-sized farm with different types of use, as commonly found in Brazil. The abundance and mass of plastic debris were spatially heterogeneous, ranging from 0.509 × 10^3^ to 104 × 10^3^ pieces ha^–1^ and from 0.039 to 405 kg ha^–1^, respectively.

In conclusion, these findings highlight the influence of land topography and vegetation density on the accumulation of plastic debris. Areas with gentle slopes and sparse vegetation facilitate plastic dispersion, while denser vegetation in forested areas acts as a natural barrier, retaining debris with a higher mass concentration. Our research has demonstrated that agricultural practices contribute to the fragmentation of macroplastics into smaller particles, such as mesoplastics and microplastics, which can worsen soil contamination.

The types of polymers identified in the study area were polypropylene (PP), polyethylene (PE), and poly(vinyl chloride) (PVC). However, our results indicate that contamination is largely attributed to the widespread use of PE and PP in agricultural operations. In general, this plastic debris primarily originates from materials used for plant labeling, agrochemical storage, irrigation systems, and various types of bags. Additionally, degraded plastic debris was found predominantly in agricultural areas, which exhibited the highest abundance and mass of plastic waste. In summary, our findings emphasize that agricultural practices are the main drivers of plastic debris accumulation. Therefore, effective management strategies to mitigate environmental impacts are essential.

## Supporting Information Available

Slope data; LS factor; NDVI index of the study site; and the quantity and size of plastic debris used for multiple factor analysis (Tables S1–S3) (PDF)

## Abbreviations

MaP: macroplastics
MeP: mesoplastics
GMP: coarse microplastics
MP: microplastics
PE: polyethylene
PP: polypropylene
PS: polystyrene
PVC: polyvinyl chloride
GM: gleysols
LVA: oxisols
NX: nitisols
CX: cambisols
SOM: soil organic matter
