# Supplementary material for: Plastic Debris in Agroecosystems: Distribution and Abundance Patterns, and Relationship with Terrain Characteristics in Southeastern Brazil
Source: ACS Omega. 2025 May 1;10(18):18457–69. doi: 10.1021/acsomega.4c10678 (PMC12079247; doi:10.1021/acsomega.4c10678)
Supplement: Supplementary file 1 — ao4c10678_si_001.pdf [file ao4c10678_si_001.pdf]

Supporting Information for

Plastic debris in agroecosystems: Distribution and abundance patterns, and relationship with terrain characteristics in Southeastern Brazil

*John Jairo Arévalo-Hernández [a,b] *, Angela Barrera de Brito [c], Junior Cesar Avanzi [a], Marcelo Angelo Cirillo [d], Marx Leandro Naves Silva [a]*

[a] Federal University of Lavras, Department of Soil Science, P.O. Box 3037, 37203-202 Lavras, MG, Brazil

[b] Surcolombiana University, Engineering Faculte, Avenida Pastrana Borrero, Carrera 1, 410010 Neiva, Huila, Colombia

[c] Federal University of Lavras, Department of Physics, P.O. Box 3037, 37203-202 Lavras, MG, Brazil

[d] Federal University of Lavras, Department of Statistics, P.O. Box 3037, 37203-202 Lavras, MG, Brazil.

*Corresponding author email: john.arevalo@usco.edu.co

Contents

Table S1 ………………………………………………………….. S2

Table S2…………………………………………………………… S3

Table S3…………………………………………………………… S4

Table S1: Average values of the abundance group (number and mass) and the dimension group (area, perimeter, and diameter) of plastic debris collected on the soil surface as a function of four slope classes.

Class_slope	Abundance Group		Dimension Group		
	Quantity (pieces x10^3 ha^{-1})	Mass (kg ha^{-1})	Area (m^2 ha^{-1})	Perimeter (m ha^{-1})	Diameter (m ha^{-1})
Flat	9.297	16.343	54.021	3543.976	898.599
Gently undulating	9.366	10.513	19.934	2128.444	542.859
Moderately undulating	11.717	24.809	32.126	2645.922	662.571
Undulating	17.320	0.695	5.292	1718.594	443.709

Table S2: Average values of the abundance group (quantity and mass) and the dimension group (area, perimeter, and diameter) of plastic debris collected on the soil surface as a function of seven LS factor intervals.

Class_slope	Abundance Group		Dimension Group		
	Quantity (pieces x10^3 ha^{-1})	Mass (kg ha^{-1})	Area (m^2 ha^{-1})	Perimeter (m ha^{-1})	Diameter (m ha^{-1})
[0.12684-0.81824]	15.419	15.750	26.560	2947.622	724.660
[0.81824-1.50964]	11.292	12.720	29.794	3183.372	841.919
[1.50964-2.20104]	10.507	58.594	67.789	2797.517	666.544
[2.20104-2.89244]	6.477	13.306	9.349	932.711	233.874
[2.89244-3.58384]	3.057	6.076	8.730	732.606	136.900
[3.58384-4.27524]	1.019	6.299	7.522	738.808	161.330

Table S3: Average values of the abundance group (quantity and mass) and the dimension group (area, perimeter, and diameter) of plastic debris collected on the soil surface as a function of seven NDVI index intervals.

Class_slope	Abundance Group		Dimension Group		
	Quantity (pieces x10^3 ha^{-1})	Mass (kg ha^{-1})	Area (m^2 ha^{-1})	Perimeter (m ha^{-1})	Diameter (m ha^{-1})
[0.06011-0.10591]	12.335	18.583	33.728	3514.991	830.615
[0.10591-0.15171]	15.390	9.603	26.490	3031.534	802.155
[0.15171-0.19751]	6.914	16.876	18.407	1618.361	392.364
[0.19751-0.24331]	6.028	2.735	5.392	762.798	199.266
[0.24331-0.28911]	2.802	0.419	3.336	658.591	147.972
[0.28911-0.35491]	0.509	40.560	258.584	1507.896	478.859
